# Supplementary material for: Comprehensive statistical inference of the clonal structure of cancer from multiple biopsies
Source: Sci Rep. 2017 Dec 5;7:16943. doi: 10.1038/s41598-017-16813-4 (PMC5717219; doi:10.1038/s41598-017-16813-4)
Supplement: Supplementary file 1 — Supplementary information [file 41598_2017_16813_MOESM1_ESM.pdf]

Supplement to "Comprehensive statistical inference of the clonal structure of cancer from multiple biopsies"

Jie Liu[1], John T. Halloran[2], Jeffrey A. Bilmes[2], Riza M. Daza[1], Choli Lee[1], Elisabeth M. Mahen[3,4,5], Donna Prunkard[6], Chaozhong Song[3,4,5], Sibel Blau[3,7], Michael O. Dorschner[3,6], Vijayakrishna K. Gadi[8,9], Jay Shendure[1], C. Anthony Blau[3,4,5,*] and William S. Noble[1,10,*]

[1]Department of Genome Sciences, University of Washington, Seattle, WA
[2]Department of Electrical Engineering, University of Washington, Seattle, WA
[3]Center for Cancer Innovation, University of Washington, Seattle, WA
[4]Institute for Stem Cell and Regenerative Medicine, University of Washington, Seattle, WA
[5]Department of Medicine/Hematology, University of Washington, Seattle, WA
[6]Department of Pathology, University of Washington, Seattle, WA
[7]Northwest Medical Specialties, Puyallup and Tacoma, WA
[8]Department of Medicine/Oncology, University of Washington, Seattle, WA
[9]Seattle Cancer Care Alliance, Seattle, WA
[10]Department of Computer Science and Engineering, University of Washington, Seattle, WA

[*]Correspondence to Dr. C. Anthony Blau, 850 Republican St., Box 358056, University of Washington, Seattle, WA 98109, USA (tblau@uw.edu) and Dr. William S. Noble, 3720 15th Ave NE, Box 355065, University of Washington, Seattle, WA 98195, USA (william-noble@uw.edu)

Supplementary Figures

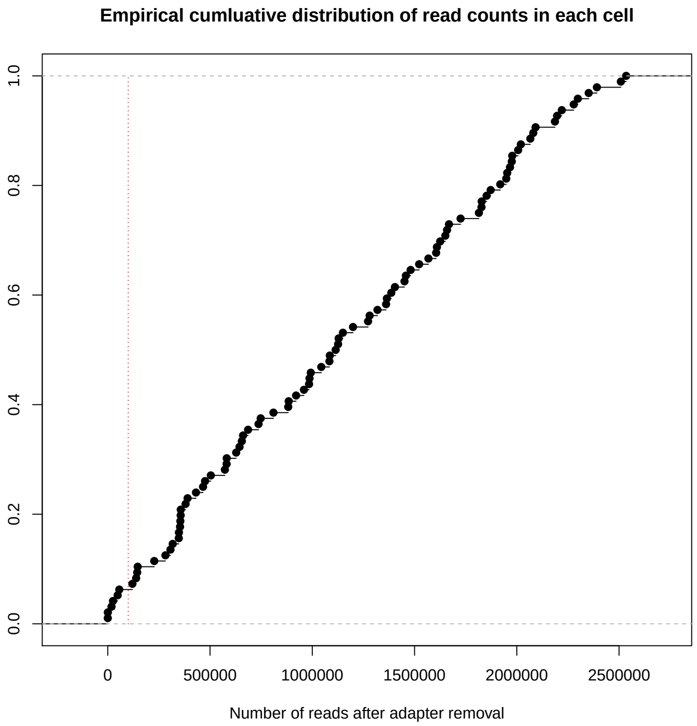

Figure 1: The empirical cumulative distribution of read counts in each cell (six cells with fewer than 100,000 reads, shown as the red dotted line, are removed).

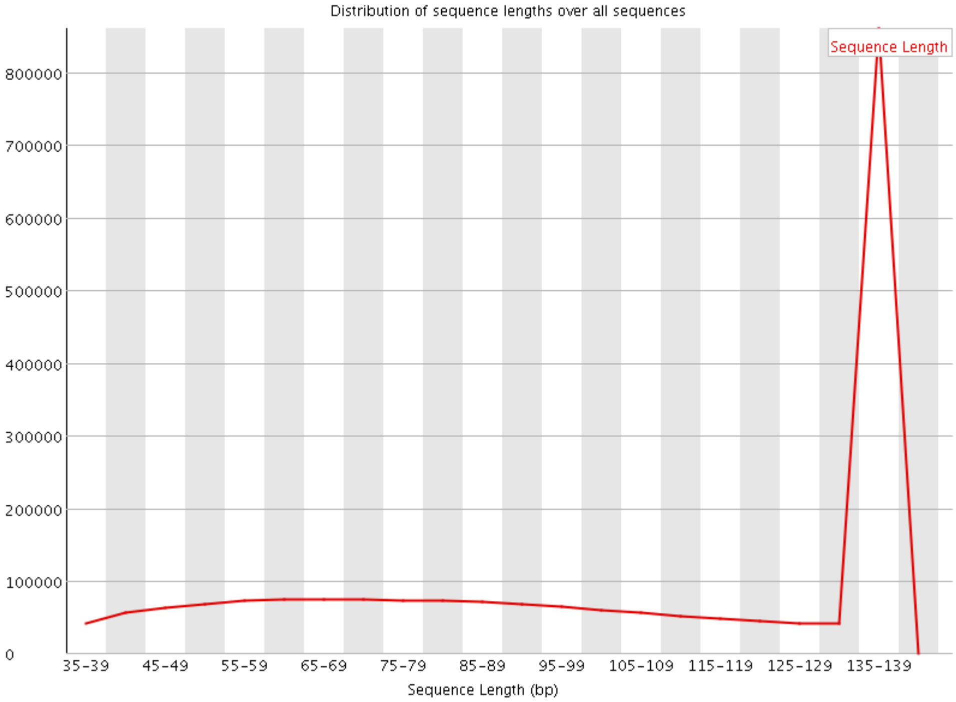

(a) Cell #28

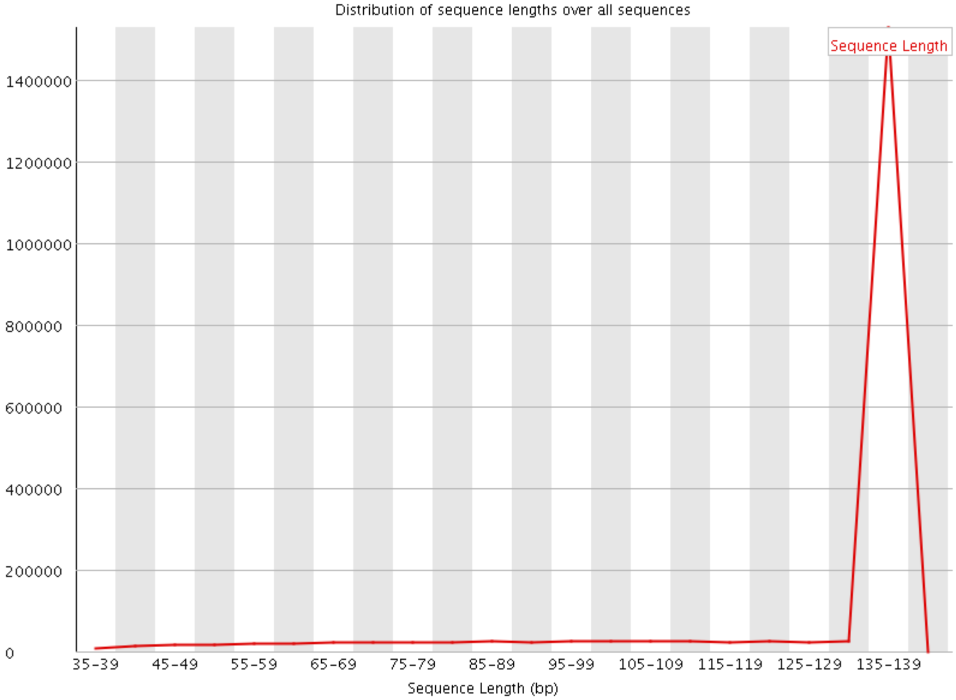

(b) Cell #1

Figure 2: A larger portion of shorter reads are observed in cell #28 than in cell #1. (a) The sequence length distribution of cell #28. (b) The sequence length distribution of cell #1.

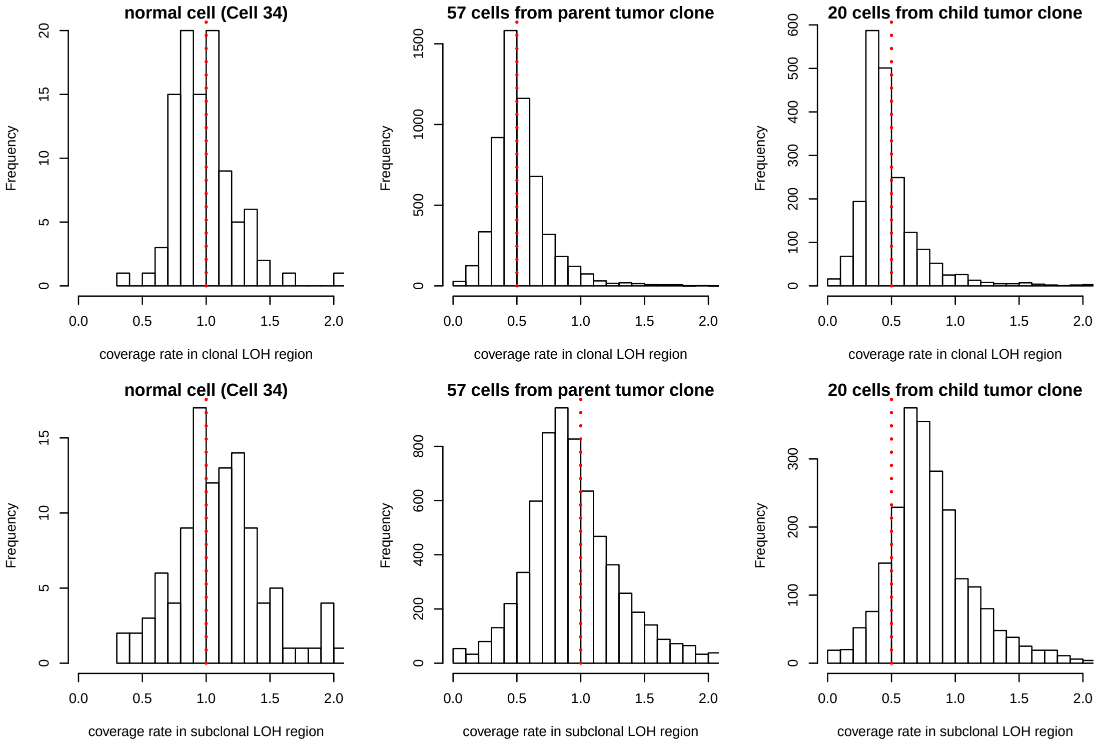

Figure 3: Histograms of the relative coverage rate in clonal LOH segments and subclonal LOH segments in a normal cell (Cell #34), 57 cells from parent tumor clone and 20 cells from child tumor clone. Red dotted lines in the histograms indicate the expected coverage rates in the cells.

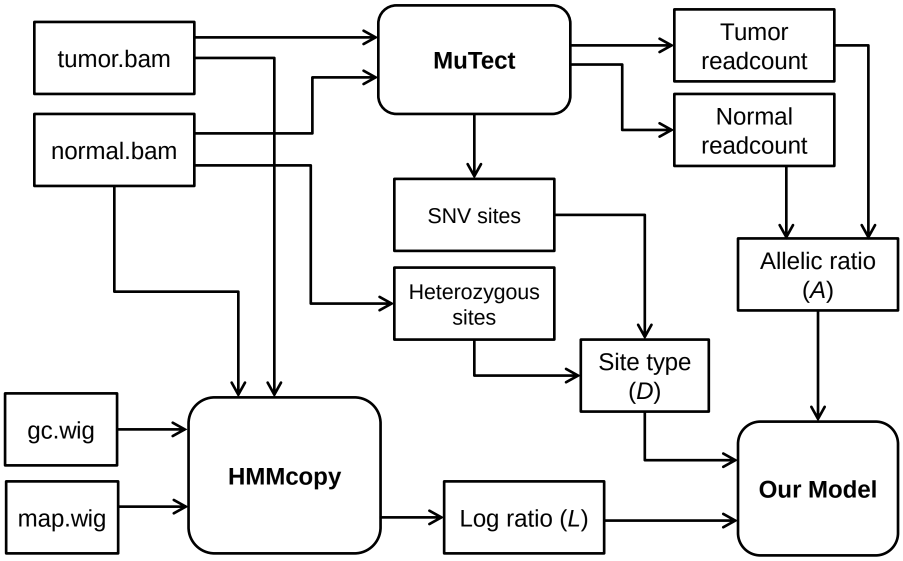

Figure 4: The data preprocessing diagram.

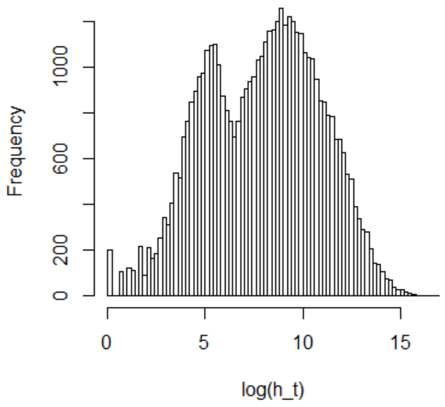

Figure 5: Histogram of $\log(\bar{h}_t))$ in one of tumor biopsies in our analysis.

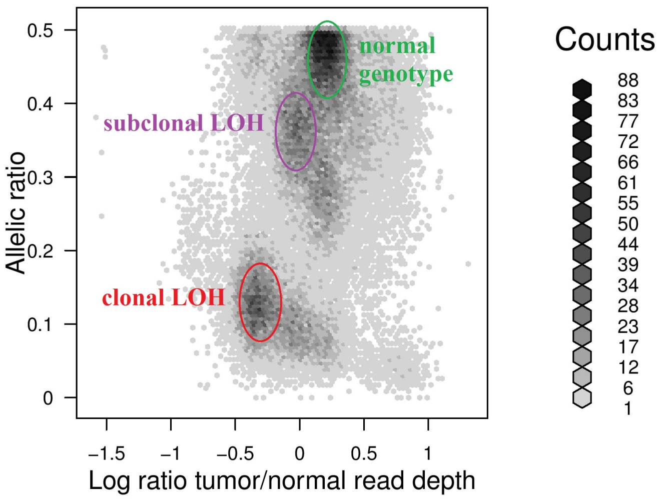

Figure 6: A bivariate plot of allelic ratio and log ratio from a tumor biopsy with subclonal LOH events and clonal LOH events annotated.

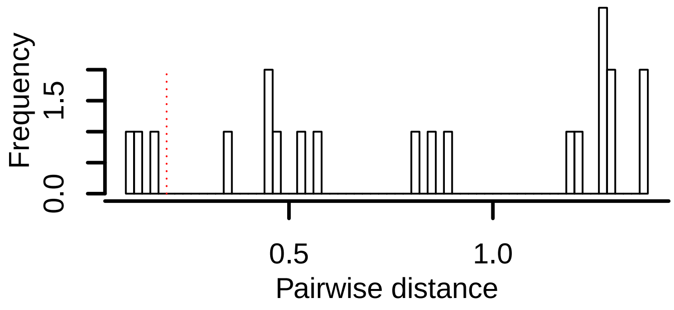

Figure 7: Histogram of the pairwise distances and the threshold (dashed red line) for merging the clones.

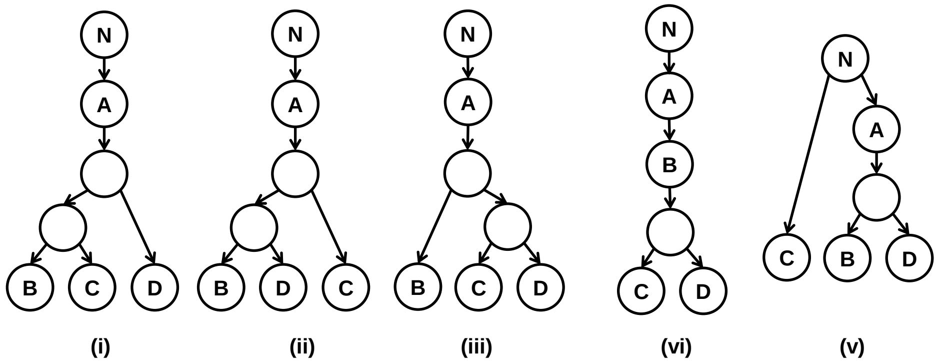

Figure 8: Candidate phylogenetic trees considered in the joint analysis on three tumor biopsies.

Supplementary Tables

Table 1: Biopsy and genomic position information used in simulations

Biopsy ID in ITOMIC study	10-1-B1	11-2-B1	12-1-B1
Location	Right breast	Right hepatic lobe	Skin punch
# germline heterozygous sites	381,776	238,304	360,772
# heterozygous sites (sequenced $\geq$ 50 times)	62,604	61,125	58,439
# SNV sites	562	484	341

Table 2: Error rates from THEMIS and TITAN in terms of genotype recovery (i.e. variable G), clonal/subclonal status recovery (i.e. variable Z), and the recovery of both genotype and clonal/subclonal status (i.e. variables G,Z) in the simulation experiments

Error rate		Experiment 1 (15% normal cells, 50% parent tumor cells, 35% child tumor cells)			Experiment 2 (25% normal cells, 45% parent tumor cells, 30% child tumor cells)			Experiment 3 (35% normal cells, 40% parent tumor cells, 25% child tumor cells)		
		10-1-B1	11-2-B1	12-1-B1	10-1-B1	11-2-B1	12-1-B1	10-1-B1	11-2-B1	12-1-B1
THEMIS on all sites	G	0.066	0.071	0.047	0.084	0.09	0.059	0.12	0.131	0.081
	Z	0.043	0.041	0.029	0.052	0.052	0.037	0.073	0.076	0.046
	G,Z	0.067	0.072	0.048	0.085	0.091	0.061	0.122	0.134	0.083
THEMIS on hetero sites	G	0.066	0.071	0.047	0.084	0.09	0.059	0.12	0.131	0.081
	Z	0.043	0.04	0.029	0.052	0.052	0.037	0.073	0.076	0.046
	G,Z	0.067	0.072	0.048	0.085	0.091	0.061	0.122	0.134	0.083
THEMIS on SNV sites	G	0.053	0.094	0.069	0.078	0.094	0.062	0.093	0.158	0.111
	Z	0.033	0.051	0.042	0.048	0.05	0.035	0.058	0.094	0.069
	G,Z	0.053	0.094	0.069	0.078	0.094	0.062	0.093	0.158	0.111
THEMIS on TITAN's sites	G	0.067	0.072	0.053	0.085	0.091	0.066	0.122	0.133	0.09
	Z	0.044	0.041	0.03	0.052	0.052	0.038	0.074	0.077	0.048
	G,Z	0.068	0.074	0.054	0.087	0.093	0.067	0.124	0.136	0.092
TITAN on TITAN's sites	G	0.077	0.082	0.076	0.096	0.101	0.089	0.126	0.135	0.109
	Z	0.074	0.077	0.074	0.092	0.094	0.085	0.121	0.127	0.103
	G,Z	0.078	0.083	0.077	0.097	0.103	0.091	0.128	0.137	0.11

Table 3: The number of segments (used for distinguishing individual cells) and the total length in different regions

Regions	Number of segments	Total length (in base pairs)
Clonal 1-copy	99	674,430,365
Clonal 2-copy	298	803,986,304
Subclonal 1-copy	119	380,786,754
total	516	1,859,203,423

Table 4: The log likelihood under normal subclone, parent tumor subclone and child tumor subclone of each of the 79 single cells used in validation experiment

cell #	l(normal)	l(parent)	l(child)	cell #	l(normal)	l(parent)	l(child)
1	-3.2004E+08	-2.9947E+08	**-2.9898E+08**	50	-7.4295E+07	**-7.1466E+07**	-7.1690E+07
3	-3.5640E+08	-3.3008E+08	**-3.2972E+08**	51	-5.1788E+07	**-5.0020E+07**	-5.0220E+07
4	-2.1836E+08	-2.0620E+08	**-2.0594E+08**	53	-7.6817E+07	**-7.4164E+07**	-7.4520E+07
5	-2.5271E+08	**-2.3832E+08**	-2.3844E+08	54	-6.0665E+07	-5.7933E+07	**-5.7791E+07**
6	-3.1863E+08	**-2.9848E+08**	-3.0229E+08	55	-8.8722E+07	**-8.5161E+07**	-8.5467E+07
7	-3.1821E+08	**-3.0256E+08**	-3.0439E+08	56	-1.0068E+08	**-9.6935E+07**	-9.7192E+07
8	-1.8316E+08	**-1.7514E+08**	-1.7664E+08	57	-1.9626E+08	**-1.8703E+08**	-1.8705E+08
9	-2.7598E+08	**-2.5913E+08**	-2.6097E+08	58	-2.4129E+08	**-2.2725E+08**	-2.2797E+08
10	-2.7347E+08	**-2.6050E+08**	-2.6249E+08	59	-1.2560E+08	**-1.1967E+08**	-1.1979E+08
11	-2.6770E+08	**-2.5156E+08**	-2.5173E+08	61	-1.6600E+08	-1.5721E+08	**-1.5652E+08**
12	-1.7124E+08	-1.5682E+08	**-1.5309E+08**	62	-1.4721E+08	**-1.3980E+08**	-1.4107E+08
13	-1.8561E+08	-1.7969E+08	**-1.7887E+08**	63	-2.2238E+08	**-2.0982E+08**	-2.0988E+08
14	-2.7827E+08	**-2.5852E+08**	-2.6094E+08	64	-1.5339E+08	**-1.4605E+08**	-1.4625E+08
15	-1.7914E+08	-1.6797E+08	**-1.6666E+08**	65	-2.1824E+08	-2.0635E+08	**-2.0543E+08**
16	-1.8871E+08	-1.7539E+08	**-1.7365E+08**	66	-1.2940E+08	-1.2171E+08	**-1.2060E+08**
17	-1.7866E+08	-1.6893E+08	**-1.6851E+08**	67	-1.2309E+08	**-1.1786E+08**	-1.1935E+08
18	-3.0793E+08	**-2.9007E+08**	-2.9081E+08	69	-1.1456E+08	**-1.0928E+08**	-1.0954E+08
19	-1.6689E+08	**-1.5882E+08**	-1.5956E+08	70	-2.7109E+08	**-2.5492E+08**	-2.5556E+08
21	-2.0313E+08	**-1.9333E+08**	-1.9467E+08	71	-3.5781E+08	**-3.3262E+08**	-3.3477E+08
22	-2.8308E+08	-2.6165E+08	**-2.6106E+08**	72	-2.6955E+08	**-2.5607E+08**	-2.5837E+08
25	-2.7853E+08	**-2.6493E+08**	-2.6660E+08	73	-7.6656E+07	**-7.3857E+07**	-7.4123E+07
26	-3.4310E+08	**-3.2498E+08**	-3.2927E+08	74	-7.8724E+07	**-7.6066E+07**	-7.6294E+07
27	**-2.7629E+08**	-2.7871E+08	-2.8475E+08	75	-7.6823E+07	**-7.4390E+07**	-7.4649E+07
29	-2.1423E+08	**-2.0372E+08**	-2.0452E+08	77	-6.9636E+07	**-6.7014E+07**	-6.7068E+07
30	-3.0135E+08	**-2.8201E+08**	-2.8206E+08	78	-1.0706E+08	-1.0217E+08	**-1.0213E+08**
31	-3.4741E+08	**-3.2217E+08**	-3.2363E+08	79	-1.5776E+08	-1.4935E+08	**-1.4916E+08**
33	-3.2762E+08	**-3.1257E+08**	-3.1617E+08	80	-4.6847E+07	-4.4739E+07	**-4.4649E+07**
34	**-3.7915E+08**	-3.9405E+08	-4.0200E+08	81	-3.0290E+08	**-2.8330E+08**	-2.8407E+08
35	-2.1701E+08	**-2.0419E+08**	-2.0520E+08	82	-2.0842E+08	-1.9738E+08	**-1.9730E+08**
37	-2.4257E+08	**-2.3316E+08**	-2.3436E+08	83	-5.9097E+07	-5.6386E+07	**-5.6228E+07**
38	-2.7988E+08	**-2.6079E+08**	-2.6197E+08	85	-1.1925E+08	**-1.1645E+08**	-1.1697E+08
39	-2.6259E+08	-2.4341E+08	**-2.4225E+08**	86	-1.7056E+08	**-1.6266E+08**	-1.6273E+08
40	-1.0023E+08	**-9.6249E+07**	-9.6685E+07	87	-1.5134E+08	**-1.4324E+08**	-1.4347E+08
41	-1.0584E+08	**-1.0136E+08**	-1.0147E+08	88	-1.8733E+08	-1.8047E+08	**-1.8012E+08**
43	-1.6028E+08	**-1.5345E+08**	-1.5413E+08	89	-7.2007E+07	**-6.8882E+07**	-6.8957E+07
45	-2.5332E+08	**-2.4075E+08**	-2.4153E+08	90	-3.8732E+07	**-3.7733E+07**	-3.7846E+07
46	-3.5165E+08	**-3.3335E+08**	-3.3741E+08	93	-7.6905E+07	**-7.4398E+07**	-7.4737E+07
47	-2.4318E+08	**-2.2891E+08**	-2.2919E+08	95	-3.3512E+07	**-3.2555E+07**	-3.2758E+07
48	-2.1306E+08	**-2.0327E+08**	-2.0424E+08	96	-1.6207E+08	**-1.5596E+08**	-1.5651E+08
49	-3.6071E+07	**-3.4976E+07**	-3.5054E+07				

Table 5: Explanation of notation, including lower and upper scripts, random variables, parameters to be estimated and hyperparameters to be pre-specified

Type	Notation	Explanation
Subscripts & superscripts	m	Index of biopsies, $m = 1,\ldots, M$
	t	Index of genomic positions/sites, $t = 1,\ldots, T$
	g	For variables related to a specific genotype g
	z	For variables related to a specific clone z
	N	For variables related to normal cells
	alt	For variables related to alternative alleles
Random variables	D_t	Type of site t, germline heterozygous site or somatic mutation site
	S_t	Whether site t is the first site on the chromosome
	H_t	The distance (in base pairs) between site t and its previous site $t-1$
	G_t	Genotype at site t
	Z_t	Index of the clone to which the somatic event at site t happens
	P_m^z	Cell prevalence of clone z in biopsy m
	$A_{m,t}$	Allelic ratio at site t in biopsy m
	$L_{m,t}$	Log ratio between tumor and normal read depth at site t in biopsy m
Parameters to be estimated	$\sigma^2_{A,m,0}$	Variance of allelic ratio in biopsy m on germline heterozygous sites
	$\sigma^2_{A,m,1}$	Variance of allelic ratio in biopsy m on somatic mutation sites
	$\sigma^2_{L,m}$	Variance of log ratio in biopsy m on each site
	Q^G	Transition probability between genotypes
	Q^Z	Transition probability between clones
	$\rho_G(j; \bar{h}_t)$	Probability of staying at the same genotype j if previous site is $\bar{h}_t$ base pairs away
	$\rho_Z(j; \bar{h}_t)$	Probability of staying at the same clone j if previous site is $\bar{h}_t$ base pairs away
	$\sigma^2_{G,j}$	Parameter in $\rho_G(j; \bar{h}_t)$, $\forall j \in 1, \ldots, \lvert P\rvert$
	$\sigma^2_{Z,j}$	Parameter in $\rho_Z(j; \bar{h}_t)$, $\forall j \in 1, \ldots, \lvert Z\rvert$
	π_G	Prior distribution of genotypes
	π_Z	Prior distribution of clones
	π_P	Prior distribution of prevalence levels
Parameters to be pre-specified	c^T_{max}	Maximum copy number in CNA events in tumor cells, 5 by default
	$\lvert Z\rvert$	Number of clones
	$\lvert P\rvert$	Number of prevalence levels
	c_m	Log-ratio offset in biopsy m due to ploidy and sequencing depth change

Table 6: All possible genotypes (up to 5 copies) and their corresponding copy number (n_G), the number of alternative alleles (n_G^{alt}) and the call [1] (colored the same way as our figures in the paper)

State	Genotype	n_g	n_g^{alt}	Call
0	NA	0	0	HOMD (homozygous deletion)
1	A or B	1	0	DLOH (hemizygous deletion)
2	AA or BB	2	0	NLOH (copy neutral LOH)
3	AB	2	1	HET (diploid heterozygous)
4	AAA or BBB	3	0	ALOH (amplified LOH)
5	AAB or ABB	3	1	GAIN (gain/duplication of 1 allele)
6	AAAA or BBBB	4	0	ALOH (amplified LOH)
7	AAAB or ABBB	4	1	ASCNA (allele-specific copy number amplification)
8	AABB	4	2	BCNA (balanced copy number amplification)
9	AAAAA or BBBBB	5	0	ALOH (amplified LOH)
10	AAAAB or ABBBB	5	1	ASCNA (allele-specific copy number amplification)
11	AAABB or AABBB	5	2	UBCNA (unbalanced copy number amplification)

Table 7: Identifying the number of clones via a naive eyeball method, Bayesian information criterion and cross-validation in simulations and a tumor biopsy (numbers with * are from approximate runs, ck-beam=2,000,000)

	Eyeball	Bayesian information criterion			avg. $\ln L$ in CV		
		2-clone	3-clone	4-clone	2-clone	3-clone	4-clone
Simulation 1	2 subclones	**-114,460**	-110,740	–	**19,078**	18,496	–
Simulation 2	3 subclones	-105,370	**-109,778**	-98,069*	17,306	**18,372**	16,079*
Tumor biopsy	2 subclones	**-476,816**	-464,822	–	**79,453**	77,426	–

Table 8: Identifying the numbers of clones in the three biopsies via cross validation (CV) (because the 2-Clone models have higher CV log likelihood than the 3-Clone models, we assume that each biopsy has two tumor clones)

Biopsies	CV log-L (2-Clone)	CV log-L (3-Clone)
B1	**79,452.68**	77,452.65
B2	**117,716.07**	115,219.87
B3	**81,698.01**	79,674.50

Table 9: The estimated prevalences of the clones in the three biopsies

Biopsies	Clone 1 (C1)	Clone 2 (C2)
B1	75%	35%
B2	45%	20%
B3	70%	30%

Table 10: The pairwise distance matrix among the clones based on their genome-wide genotype (the distances below the threshold are shown in bold)

	Normal	B1-C1	B1-C2	B2-C1	B2-C2	B3-C1	B3-C2
Normal	0	0.45	0.812	0.523	0.855	0.471	0.892
B1-C1	0.45	0	1.262	**0.123**	1.285	**0.108**	1.3
B1-C2	0.812	1.262	0	1.186	0.349	1.207	0.451
B2-C1	0.523	**0.123**	1.186	0	1.378	**0.176**	1.269
B2-C2	0.855	1.285	0.349	1.378	0	1.267	0.567
B3-C1	0.471	**0.108**	1.207	**0.176**	1.267	0	1.363
B3-C2	0.892	1.3	0.451	1.269	0.567	1.363	0

Table 11: The distance among the four tumor clones and the normal clone based on genome-wide genotype after identifying parent-child relationship within each biopsy

	Normal	A	B	C	D
Normal	0.000	0.481	1.262	1.378	1.363
A	0.481	0.000	0.814	0.928	0.924
B	1.262	0.814	0.000	0.303	0.440
C	1.378	0.928	0.303	0.000	0.538
D	1.363	0.924	0.440	0.538	0.000

Table 12: The numbers of genes with copy number changes during the different stages of cancer progression in important cancer signaling pathways. * denotes at least one of the mutated genes is a core component of the signaling pathway.

	B→CD		CD→C			CD→D		
	35 CNAs<br>34/208 genes relevant		112 CNAs<br>152/636 genes relevant			98 CNA's<br>175/519 genes relevant		
	LOH<br>b	Gain<br>abb	LOH<br>b	LOH<br>bb	Gain<br>abb	LOH<br>b	Gain<br>abb	Gain<br>aabb
Rho/rab/ras	3	1	7	1	6	6	12*	1
MEK/MAPK/Erk	2	0	4	1	1*	2	14	0
JNKs/JUN	1	0	2	0	1	1	4	0
Myc	1	2	1	0	2	0	2	1
PI3K/Akt/mTor, PTEN	1	5*	6	0	6*	3	14*	0
PKC	0	0	0	1	2	0	0	0
NF-kappaB	0	4	4	1	7	2	12	0
JAK/STAT	1	1	5	0	4	2	2	0
apoptosis/caspase	1	1	2	0	1	1	8	1
Bcl-2	0	0	3	0	2	2	1	0
TNF/TNF-alpha	0	3	0	0	0	1	3*	0
ERBB	0	0	0	0	3	0	0	0
egf/egfr	0	0	3	0	0	0	3	0
Hippo/Yap	0	0	1	1	1	1	1	0
Wnt/beta-catenin	0	1	7	0	3	2	5	0
GSK3beta	0	0	0	0	1	1	2*	0
notch	1	0	0	0	0	0	1	1*
hedgehog	0	0	0	0	1	0	1	0
TGF-beta	2	0	4	0	4	2	5	0
Dna damage/repair	0	0	1	1	5	4	6	0
cell cycle/cyclin	1	0	5	1	1	2	6	0
angiogenesis/VEGF	0	0	3	0	0	1	3	0
p53	1	1	5	1*	4	2	14	0
MET	0	0	0	0	0	0	1	1
brca1	1	0	1	0	1	0	1	0
FGFR	0	0	0	0	0	0	1	0
cdc42	0	0	2	0	4	1	1	0
hsp70	0	0	1	1	1	0	1	0
hsp90	0	0	0	0	1	1	1	0
RAD51	0	0	0	0	0	1	1	0
AR	0	0	0	0	0	0	1	0
cox2	0	0	0	0	0	2	0	0
pka	0	1	0	0	0	1	0	1
**sum**	16	20	67	9	62	41	127	6

Table 13: Signaling pathways and their relevant genes mutated in different stages of cancer progression.

Pathways	Genes (genotype) mutated in B→ CD
Rho/rab/ras	RAP2C (-B), RIN2 (ABB), DOK1 (-B), RTKN (-B)
MEK/MAPK/Erk	MST4 (-B), TACR1 (-B)
JNK/JUN	MINA (-B)
myc	MINA (-B), ATAD3A (ABB), PNPT1 (ABB)
PI3K/Akt/mTor	DVL1 (ABB), MXRA8 (ABB), GNB1 (ABB), TACR1 (-B), SMG1 (ABB), PIK3C2A (ABB)
NF-kappaB	MIB2 (ABB), CSE1L (ABB), NPPA (ABB), NFKBIZ (ABB)
JAK/STAT	MINA (-B), TYK2 (ABB)
apoptosis/caspase	HTRA2 (-B), ARL6IP1 (ABB)
TNF/TNF-alpha	TNFRSF4 (ABB), TNFRSF1B (ABB), TNFRSF8 (ABB)
Wnt/beta-catenin	ARFGEF2 (ABB)
notch	HS6ST2 (-B)
TGF-beta	MINA (-B), TRA (-B)
cell cycle/cyclin	FHL1 (-B)
p53	MAGEA2 (-B), MDM2 (ABB)
brca1	POU1F1 (-B)
pka	ARFGEF2 (ABB)
	**Genes (genotype) mutated in CD → C**
Rho/rab/ras	RAB33A (-B), RHPN2 (ABB), CTSS (ABB), PI4KB (ABB), RAB40A (-B),PAK3 (-B), OPHN1 (-B), STARD8 (-B), RASSF3 (-B), PLXNB3 (BB), ZC3H10 (ABB), RABGGTB (-B), ARHGEF18 (ABB), RASSF2 (ABB)
MEK/MAPK/Erk	DUSP23 (-B), ALCAM (-B), DIRAS3 (-B), UBE2C (BB), MAP2K7 (ABB), IL12A (-B)
JNK/JUN	HIPK3 (ABB), DEPDC1 (-B), ELF4 (-B)
myc	RPL5 (ABB), EIF4G1 (ABB), MYCN (-B)
PI3K/Akt/mTor	PREX1 (-B), ALCAM (-B), CBLB (-B), PIK3CD (ABB), PI4KB (ABB), TRIB2 (-B), PPBP (-B), IRS4 (-B), GLS2 (ABB), CCDC88A (ABB)
PTEN	MAST3 (ABB), RPL22L1 (ABB)
PKC	SDC4 (BB), PRKCZ (ABB), PRKCI (ABB)
NF-kappaB	BST2 (ABB), CHEK1 (ABB), SLPI (BB), ARNT (ABB), MLLT11 (ABB), PSMD4 (ABB), LDOC1 (-B), CAPN6 (-B), EDA2R (-B), ATRAID (ABB), ZC3H10 (ABB), C20orf26 (-B)
JAK/STAT	REG3A (-B), REG1B (-B), REG1A (-B), REG3G (-B), JAK3 (ABB), SALL4 (-B), IL23A (ABB), STAT2 (ABB), BIRC6 (ABB)
apoptosis/caspase	CERS2 (ABB), KANK1 (-B), GZMH (-B)
Bcl-2	BNIPL (ABB), MCL1 (ABB), FATE1 (-B), CRYZ (-B), MAGED1 (-B)
ERBB	ERBB3 (ABB), PA2G4 (ABB), RNF41 (ABB)
egf/egfr	AREG (-B), EPGN (-B), EREG (-B)
Hippo/Yap	OR10G2 (-B), ANKRD52 (ABB), C20orf27 (BB)
Wnt/beta-catenin	SULF2 (-B), CITED1 (-B), CUL4B (-B), WLS (-B), CTNNBIP1 (ABB), ECM1 (ABB), SETDB1 (ABB), BCL9 (-B), WIF1 (-B), SMARCA1 (-B)
GSK3beta	CTNNBIP1 (ABB)
hedgehog	CDON (ABB)
TGF-beta	TRA (-B), SPSB1 (ABB), FCRL4 (-B), GDF7 (-B), KLF15 (-B), EIF5A2 (ABB), SKIL (ABB), ZNF217 (ABB)
Dna damage and repair	C19orf40 (ABB), INTS3 (ABB), ANKRD52 (ABB), TIMELESS (ABB), SMEK2 (ABB), SSX2 (-B), DDX11 (BB)
cell cycle/cyclin	BBX (-B), CMTM5 (-B), USH1C (-B), RPL41 (ABB), ITM2A (-B), HOXC10 (-B), NINL (BB)
angiogenesis/VEGF	C1GALT1C1 (-B), AMOT (-B), XAGE1B (-B)
p53	HDAC8 (-B), MCTS1 (-B), ZBTB33 (-B), UBE4B (ABB), TP53TG5 (BB), EDA2R (-B), CGREF1 (ABB), CNPY2 (ABB), GLS2 (ABB), TAF9B (-B)
brca1	GADD45A (-B), NABP2 (ABB)
cdc42	ARHGAP36 (-B), DOCK6 (ABB), BNIPL (ABB), CDC42SE1 (ABB), SRGAP1 (-B), TRIP10 (ABB)
hsp70	AIFM1 (-B), TIMM44 (ABB), HSPA12B (BB)
hsp90	TOMM34 (ABB)
	**Genes (genotype) mutated in CD → D**
Rho/rab/ras	RASAL3 (AABB), ATP6V1A (-B), CAPRIN1 (ABB), RASA2 (ABB), RAP1B (ABB), ARHGEF39 (-B), RUSC2 (-B), RABL3 (ABB), RHOQ (-B), ARHGAP31 (ABB), NRAS (ABB), PHF8 (ABB), RRAGB (ABB), RASSF6 (-B), MCCC1 (ABB), RASGRP3 (ABB), RGP1 (-B), RAB3IP (ABB), DENND1C (ABB)
MEK/MAPK/Erk	CHI3L2 (ABB), SPSB4 (ABB), IRAK3 (ABB), FSTL1 (ABB), RSPH3 (ABB), PDCD10 (ABB), GATA2 (ABB), MAGI3 (ABB), LY9 (ABB), STAMBP (ABB), DUSP11 (ABB), DUSP9 (-B), S100A9 (ABB), LAMP3 (ABB), TUBB4A (ABB), PDE4B (-B)
JNK/JUN	RBP1 (ABB), DYRK2 (ABB), DUSP9 (-B), TNIK (ABB), DNM2 (ABB)
myc	BRD4 (AABB), ELF5 (ABB), DYRK2 (ABB)
PI3K/Akt/mTor	CCL25 (ABB), PIK3CB (ABB), RBP1 (ABB), GSK3B (ABB), PIK3C2A (ABB), SLC16A4 (-B), RARRES1 (-B), ITLN1 (ABB), LY9 (ABB), PRR5L (ABB), ACAP2 (ABB), TNK2 (ABB), SNAI1 (-B)
PTEN	GSK3B (ABB), ABTB1 (ABB), MCM2 (ABB), USP13 (ABB)
NF-kappaB	FREM1 (ABB), KLF8 (ABB), TSR2 (ABB), COMMD9 (ABB), XIAP (-B), S100A9 (ABB), S100A7 (ABB), PLEKHG5 (ABB), TNFRSF25 (ABB), RNF114 (ABB), NKAP (ABB), BEST3 (ABB), COMMD8 (ABB), UBE2V1 (-B)
JAK/STAT	IL26 (ABB), SOCS5 (-B), HES3 (ABB), FNDC3B (-B)
apoptosis/caspase	AKAP8 (AABB), APIP (ABB), ZBTB38 (ABB), SSR3 (ABB), MIR98 (ABB), PAGE5 (ABB), SPIN2 (ABB), ZNF253 (ABB), RERE (ABB), HINT2 (-B)
Bcl-2	BCL2L15 (ABB), HOXC6 (-B), UBE2V1 (-B)
TNF/TNF-alpha	SLC9A8 (ABB), ADORA3 (-B), TNFSF9 (ABB), TNFSF14 (ABB)
egf/egfr	ELF5 (ABB), RBP1 (ABB), GATA2 (ABB)
Hippo/Yap	RMRP (-B), ITCH (ABB)
Wnt/beta-catenin	ELAVL1 (ABB), RMRP (-B), PLD1 (ABB), TNIK (ABB), S100A6 (ABB), TMEM8B (-B), MUC4 (ABB)
GSK3beta	GSK3B (ABB), PHF8 (ABB), SNAI1 (-B)
notch	NOTCH3 (AABB), ICMT (ABB)
hedgehog	DCUN1D1 (ABB)
TGF-beta	NFIB (ABB), TGFBR3 (-B), USP15 (ABB), FNDC1 (ABB), NGF (ABB), BCAR3 (-B), ACTG2 (ABB)
Dna damage and repair	CDC7 (-B), XAB2 (ABB), APEX2 (ABB), USP51 (ABB), GTSF1 (-B), SMUG1 (-B), TOPBP1 (ABB), SMC6 (-B), CHD5 (ABB), RNF113A (ABB)
cell cycle/cyclin	CCNL1 (ABB), MDM1 (ABB), GNL3L (ABB), MAGED2 (ABB), HNRNPA1 (-B), HOXC8 (-B), ELTD1 (ABB), CARM1 (ABB)
angiogenesis/VEGF	RBP2 (ABB), PROK1 (-B), PRND (ABB), TSPAN8 (ABB)
p53	EHF (ABB), NAT10 (ABB), GFI1 (-B), MIRLET7I (ABB), SIN3B (ABB), TSPAN2 (ABB), ADRA1D (ABB), RUVBL1 (ABB), HIPK1 (ABB), ZNF385A (-B), TP73 (ABB), TPRKB (ABB), S100A7 (ABB), EI24 (ABB), RNF114 (ABB), YEATS4 (ABB)
MET	SPSB4 (ABB), SPARCL1 (AABB)
brca1	SMARCA4 (ABB)
FGFR	FRS2 (ABB)
cdc42	FNBP1L (-B), TNK2 (ABB)
hsp70	DCLRE1B (ABB)
hsp90	PNCK (-B), CCT2 (ABB)
RAD51	HELB (ABB), GEN1 (-B)
AR	SLC16A7 (ABB)
cox2	LACRT (-B), MIG7 (-B)
pka	AKAP8L (AABB), DR1 (-B)

Supplementary Notes

Supplementary Note 1: Simulation details

Selecting Genomic Positions In the simulations, we used the genomic positions measured on three real tumor biopsies (biopsy ID's 10-1-B1, 11-2-B1 and 12-1-B1 in the ITOMIC study) from three patients with triple negative breast cancer (Subjects 10, 11 and 12 in the ITOMIC study). One normal (blood) biopsy from each patient was also collected and sequenced with whole exome sequencing (sequencing depths of the three normal biopsies are 231, 195 and 205) to identify germline heterozygous sites. In our simulations, we used the genomic positions at which at least 50 reads from the whole exome sequencing have been aligned. We also used the SNV sites identified by MuTect [2]. Detailed information about the biopsies and the genomic positions is provided in **Supplementary Table 1**.

Simulating genotypes and clonal/subclonal status For each genomic position, we first simulated the hidden genotype (the G variable) and the clonal/subclonal status (the Z variable) . For the G variable, we simulated the 12 genotypes listed in **Supplementary Table 6** (i.e., $G_t = 0, \ldots, 11$ at site t). For the Z variable, we assigned the somatic event at site t to be either clonal (i.e., $Z_t = 0$) or subclonal (i.e., $Z_t = 1$). For the first site on each chromosome, G and Z are drawn from a uniform distribution. For the remaining sites on each chromosome, G and Z are drawn sequentially based on the simulated xvalues at the previous site according to the transition probabilities in TITAN [3, 1]. Let d be the distance between site $t-1$ and site t (in base pairs). Define L_G to be the expected length of a CNA event, a pre-specified parameter [1]. Similarly, define L_Z to be the expected length of a CNA event in a clone [1]. Then the probability of staying at the same CNA genotype state ρ_G is [1]

$$\rho_G = 1 - [1 - \exp\{-d/(2 \times L_G)\}]/2. \tag{1}$$

The probability of staying at the same clone ρ_Z is [1]

$$\rho_Z = 1 - [1 - \exp\{-d/(2 \times L_Z)\}]/2. \tag{2}$$

The probability of CNA genotype state transition from state $i \in G$ at site $t-1$ to state j at site t, $A_t(i, j)$ is [1]

$$A_t(i,j) = \begin{cases} \rho_G, & i = j \\ \frac{1-\rho_G}{|G|-1}, & \text{otherwise.} \end{cases} \tag{3}$$

The probability of clone index transition from state $i \in Z$ at site $t-1$ to state j at site t, $T_t(i, j)$ is [1]

$$T_t(i,j) = \begin{cases} \rho_Z, & i = j \\ \frac{1-\rho_Z}{|Z|-1}, & otherwise. \end{cases} \tag{4}$$

In the simulations, L_G and L_Z were both set to 100,000. After we simulated the clonal/subclonal status at each site, we set the prevalence of the clonal events (a.k.a., tumor purity) as well as the prevalence of subclonal events. These percentages of normal cells, parent tumor cells and child tumor cells, for the three simulations are {15%, 50%, 35%}, {25%, 45%, 30%}, and {35%, 40%, 25%}, respectively.

Simulate the total number of reads in tumor biopsies Based on the simulated genotype and the simulated clonal/subclonal status at each genomic position t, we simulated the log ratio between tumor and normal read depth (the "L" variable) from a Gaussian distribution, i.e. $l_t \sim N(\mu_{g,z}, \sigma^2_{g,z})$. The mean parameter $\mu_{g,z}$ is

$$\mu_{g,z} = \log \frac{P^z c^N + (1 - P^z) c_g^T}{P^0 c^N + (1 - P^0)\phi_m}, \tag{5}$$

where c^N is the copy number in normal cells, c_g^T is the copy number in the tumor cells with genotype g, and ϕ_m is the average ploidy in tumor cells. The value of $\sigma_{g,z}$ was set to be 0.10. With the total number of reads at site t in the real normal biopsy and the simulated log-ratio value l_t, we calculated the total number of reads at site t in the tumor biopsy (rounded to the closest integer), denoted by N_t.

Simulating the total number of reads with the alternative allele With the simulated total number of reads in the tumor biopsy N_t at site t, we further simulated the number of reads with the alternative allele (N_t^B) from a binomial distribution $Bin(p_t^B, N_t)$. The "probability of success" parameter in the binomial distritbution is

$$p_t^B = \frac{n_{B,t}^T P^{Z_t} + n_{B,t}^N (1 - P^{Z_t})}{n_{A,t}^T P^{Z_t} + n_{A,t}^N (1 - P^{Z_t}) + n_{B,t}^T P^{Z_t} + n_{B,t}^N (1 - P^{Z_t})}, \tag{6}$$

where $n_{A,t}^T$ is the copy number of allele A in the cells with the somatic event at site t, $n_{A,t}^N$ is the copy number of allele A in the cells without the somatic event at site t, $n_{B,t}^T$ is the copy number of allele B in the cells with the somatic event at site t, and $n_{B,t}^N$ is the copy number of allele B in the cells without the somatic event at site t.

Algorithms We applied THEMIS and TITAN to the simulated data. Because TITAN only works on germline heterozygous sites, we removed the read counts for SNV sites when we were running TITAN. We set the parameters L_G and L_Z to be the ground truth values used in the data generation process (i.e., $L_G = L_Z =$ 100,000). For THEMIS, we also set the c parameter to be the ground truth value yielded by the data generation process. The ground truth value of c is calculated as follows. If the average ploidy in the simulated tumor biopsy is ψ and we assume that the total number of reads in the tumor biopsy is the same as the total number of reads in the normal biopsy, then at the genomic positions without CNA events, the log ratio between tumor and normal read depth (i.e., c) is $\log \frac{2.0}{\psi}$. For both THEMIS and TITAN, we also set the number of subclones and the number of possible genotypes to be the correct numbers used in the data generation process.

Performance measure We compared the Viterbi paths (the most probable states for the hidden variables G and Z) inferred from THEMIS and TITAN. Since we have two hidden variables in total, we used three performance measures. We evaluated (1) the percentage of sites at which the hidden genotype was incorrectly inferred, (2) the percentage of sites at which the clonal/subclonal status was incorrectly inferred, and (3) the percentage of sites at which either the genotype or clonal/subclonal status were incorrectly inferred. Since our algorithm analyzes both the germline heterozygous sites and SNV sites, we report the overall performance as well as the performance on germline heterozygous sites and the performance on SNV sites, respectively. TITAN only inferred the hidden variables for germline heterozygous sites at which the total number of reads aligned are between 50 and 300. Therefore, we also reported THEMIS' performance on these sites for a fair comparison with TITAN.

Supplementary Note 2: Bayesian classifier to classify single nuclei

For a segment i, we define $c_i = \sum_{k \in I_i} n_k$ to be its coverage, where n_k is the number of reads aligned to the genomic position k, and I_k is the set of positions in segment i. Let l_i be the length of segment i. We assume that there exists a coverage rate parameter, denoted by p_i, and that c_i follows a binomial distribution $bin(l_i, p_i)$. For a given p_i, the likelihood of observing c_i is

$$L(c_i; p_i) = p_i^{c_i} (1 - p_i)^{l_i - c_i}. \tag{7}$$

We use superscripts to denote the coverage rates in different regions; i.e., p^{c2}, p^{c1} and p^{sc1} are the coverage rates for clonal 2-copy region, clonal 1-copy region and subclonal 1-copy region, respectively. No matter from which subclone the cell comes, p^{c2} can be estimated unambiguously, denoted by $\hat{p}^{c2}$, because the clonal 2-copy region is the copy number unchanged region. For normal cells, $p^{c1} = p^{c2}$ and $p^{sc1} = p^{c2}$. For cells from the parent tumor subclone, $p^{c1} = 0.5 \times p^{c2}$ and $p^{sc1} = p^{c2}$. For cells from the child tumor subclone, $p^{c1} = 0.5 \times p^{c2}$ and $p^{sc1} = 0.5 \times p^{c2}$. Therefore, given a cell's coverage data $\mathbf{c} = \{c_i\}$ and coverage rate vector $\mathbf{p} = \{p^{c1}, p^{sc1}\}$, the likelihood can be calculated as

$$L(\mathbf{c}; \{p^{c1}, p^{sc1}\}) = \prod_{i \in \{clonal\ 1-copy\}} L(c_i; p^{c1}) \prod_{i \in \{subclonal\ 1-copy\}} L(c_i; p^{sc1}). \tag{8}$$

Therefore, we can calculate $L(\mathbf{c}; \textit{normal clone})$, $L(\mathbf{c}; \textit{parent tumor subclone})$ and $L(\mathbf{c}; \textit{child tumor subclone})$ for each cell, and classify (under the assumption that the prior of being each subclone is uniform) the cell to the subclone with the highest likelihood.

Supplementary Note 3: Running THEMIS on multiple biopsies from the ITO-MIC study

We first performed heterogeneity analysis on the three biopsies separately and identified two tumor clones in each biopsy (**Supplementary Table 8**) and their prevalences (**Supplementary Table 9**). We name the identified tumor clones B1-C1, B1-C2, B2-C1, B2-C2, B3-C1 and B3-C2. We then calculated the pairwise distance matrix (**Supplementary Table 10**) among the six tumor clones and the normal clone based on their inferred genotypes at the genomic positions in the model. Based on the histogram of the pairwise distance (**Supplementary Fig. 7**), we merged B1-C1, B2-C1 and B3-C1 because their pairwise distances are less than the threshold 0.2. We then identified consistent parent-child relationships based on the individually estimated prevalences (**Supplementary Table 9**). Because the sum of the prevalences of the two clones in biopsy B1 is greater than 100%, it is obvious that clones B1-C1 and B1-C2 are parent-child related. The two clones B3-C1 and B3-C2 in biopsy B3 are also assumed to be parent-child related because the sum of the prevalences of B3-C1 and B3-C2 is 100% and there should be some normal cell contamination in the biopsy. It is difficult to directly tell from the estimated prevalences in biopsy B2 whether B2-C1 and B2-C2 are parent-child related. We, for now, assume that clones B2-C1 and B2-C2 are also parent-child related because B2-C2 is quite similar to B1-C2 and B3-C2, and we have already merged B1-C1, B2-C1 and B3-C1 in the last step. For simplicity, we rename the four tumor clones *clone A* (B1-C1, B2-C1 and B3-C1), *clone B* (B1-C2), *clone C* (B2-C2) and *clone D* (B3-C2). We updated the child clones' genotype by letting them inherit their parent clones' genotype, and re-calculated the pairwise distance matrix among the clones (**Supplementary Table 11**). We then enumerated all the full phylogenies consistent with those relationships under three requirements. First, clone A is a child of the normal clone. Second, clones B, C and D are descendants of clone A. Third, clone B is not a descendant of clone C or clone D because clone B is closer to clone A compared with clone C and clone D (**Supplementary Table 11**). Structures (i), (ii) and (iii) in **Supplementary Fig. 8** are consistent with our requirements. We ran THEMIS with the three candidate structures, and the Viterbi scores (log likelihood) from the three structures are 325682.4, 325532.0 and 325779.5. When we looked into structure (iii), we noticed that there are only a few mutations on the edge connecting clone B and its parent. We suspect these mutations are artifacts, and therefore we considered another candidate structure — structure (vi) in **Supplementary Fig. 8**, in which this edge was removed. We also considered structure (v) in **Supplementary Fig. 8** in which we do not assume the two clones in biopsy B2 are parent-child related. The Viterbi scores from structures (vi) and (v) are 332194.0 and 305359.1. In the end, we selected structure (vi) as the recovered phylogenetic tree from the three biopsies.

Supplementary Note 4: Adding a constraint on the number of segments

During review of this manuscript, one reviewer suggested that the THEMIS model likely over-segments the genome. We verified this effect empirically and then demonstrated the flexibility of the THEMIS framework by modifying the model to incorporate a user-specified constraint on the number of segments.

	Experiment 1 (15% normal cells, 50% parent tumor cells, 35% child tumor cells)			Experiment 2 (25% normal cells, 45% parent tumor cells, 30% child tumor cells)			Experiment 3 (35% normal cells, 40% parent tumor cells, 25% child tumor cells)		
Biopsy	10-1-B1	11-2-B1	12-1-B1	10-1-B1	11-2-B1	12-1-B1	10-1-B1	11-2-B1	12-1-B1
True # segments	3096	2926	3281	3143	2967	3294	3204	3029	3302
TITAN # segments	3140	2985	3167	3191	2990	3186	3241	3057	3204
TITAN difference	-44	-59	114	-48	-23	108	-37	-28	98
THEMIS # segments	4039	3894	4342	4227	3886	4392	4349	3992	4592
THEMIS difference	-943	-968	-1061	-1084	-919	-1098	-1145	-963	-1290

Table 14: The table lists, for the simulations reported in the main paper, the number of segments in the simulated data, and the number of segments inferred by TITAN and THEMIS. The "difference" rows indicate when a method over-segments (negative values) or under-segments (positive values).

We first compared the tendency of TITAN and THEMIS to under- or over-segment in our simulation study. In TITAN, the number of segments is under user control. Specifically, by tuning the expected segment length parameters (L_G and L_Z), the user can obtain more or fewer segments. In the simulations reported in the subsection "Simulation results", we set the TITAN parameters L_G and L_Z to be equal to the ground truth values used in the data generation process (i.e., $L_G = L_Z =$ 100,000). In this case, we observed a mixture of over- and under-segmentation, depending upon the biopsy used for the simulation (Table 14). In contrast, THEMIS estimates L_G and L_Z from the data, rather than requiring the user to set these parameters. In this approach, the reviewer is correct that THEMIS systematically yields more segments than ground truth (Table 14). Most of the extra segments arise because THEMIS interprets a clonal "ABB" event as a subclonal "ABBB" event or a subclonal "ABBBB" event, and vice versa. This behavior arises because, in the presence of a substantial amount of sequencing noise, it is quite difficult to distinguish a clonal "ABB" event from a subclonal "ABBB" or "ABBBB" event based on the simulated A and L variables.

THEMIS		Experiment 1 (85%, 35%)			Experiment 2 (75%, 30%)			Experiment 3 (65%, 25%)		
		10-1-B1	11-2-B1	12-1-B1	10-1-B1	11-2-B1	12-1-B1	10-1-B1	11-2-B1	12-1-B1
on all sites	G	0.0658	0.071	0.0471	0.0837	0.0898	0.0592	0.12	0.131	0.0814
	Z	0.0429	0.0405	0.0291	0.0516	0.0519	0.037	0.0732	0.076	0.0464
	G,Z	0.0669	0.0724	0.048	0.0852	0.0913	0.0606	0.122	0.134	0.0832
on hetero sites	G	0.0659	0.0709	0.047	0.0837	0.0897	0.0592	0.12	0.131	0.0813
	Z	0.043	0.0405	0.0291	0.0517	0.0519	0.037	0.0734	0.0759	0.0464
	G,Z	0.067	0.0723	0.0479	0.0853	0.0913	0.0606	0.122	0.134	0.0831
on SNV sites	G	0.053	0.0942	0.0694	0.0779	0.0935	0.0625	0.0927	0.158	0.111
	Z	0.0328	0.0507	0.0417	0.0477	0.0504	0.0347	0.0576	0.0935	0.0694
	G,Z	0.053	0.0942	0.0694	0.0779	0.0935	0.0625	0.0927	0.158	0.111
**Revised Model**		**Experiment 1 (85%, 35%)**			**Experiment 2 (75%, 30%)**			**Experiment 3 (65%, 25%)**		
		10-1-B1	11-2-B1	12-1-B1	10-1-B1	11-2-B1	12-1-B1	10-1-B1	11-2-B1	12-1-B1
on all sites	G	0.049	0.0539	0.0472	0.0694	0.0731	0.0525	0.108	0.121	0.0789
	Z	0.0379	0.0373	0.0332	0.0512	0.0494	0.0373	0.0724	0.0758	0.0515
	G,Z	0.0493	0.0547	0.0478	0.0699	0.0738	0.0533	0.109	0.122	0.08
on hetero sites	G	0.0491	0.0539	0.0472	0.0695	0.0731	0.0525	0.108	0.121	0.0788
	Z	0.038	0.0374	0.0331	0.0512	0.0493	0.0373	0.0725	0.0757	0.0514
	G,Z	0.0494	0.0547	0.0478	0.0699	0.0737	0.0533	0.109	0.122	0.08
on SNV sites	G	0.0354	0.0507	0.0764	0.0678	0.101	0.0556	0.108	0.173	0.104
	Z	0.0253	0.0217	0.0625	0.0427	0.0791	0.0347	0.0677	0.108	0.0694
	G,Z	0.0354	0.0507	0.0764	0.0678	0.101	0.0556	0.108	0.173	0.104

Table 15: Error rates from THEMIS and the revised model in terms of genotype recovery (i.e. variable G), clonal/subclonal status recovery (i.e. variable Z), and the recovery of both genotype and clonal/subclonal status (i.e. variables G,Z) in the simulation experiments. Since the models analyze both the germline heterozygous sites and SNV sites, we report the overall performance as well as the performance on germline heterozygous sites and the performance on SNV sites, respectively.

To address this over-segmentation issue, we experimented with a modified version of THEMIS. During the inference of the most probable state of the hidden variables, we added another variable "seg_count", which limits the total number of segments allowed after the current frame. The variable "seg_count" is an observed variable in the first frame (i.e., the total number of segments allowed in the entire data, specified by the user) and is a hidden variable in the remaining frames. The value of "seg_count" decreases by one if a new segment is created at the current frame (e.g., either the state of G or the state of Z differs from previous frame). Once "seg_count" reaches zero, no new segment is allowed thereafter. In essence, the new model recovers the most probable state of the hidden variables under the constraint that the inferred segment number is smaller than a specified number. This revision was easily accomplished in the GMTK extensible modeling framework, thereby demonstrating the inherent flexibility of THEMIS. We applied the revised model to the previously simulated data, with "seg_count" set to be the true segment number $\times$ 1.05 (rounded to the nearest integer). We did not set "seg_count" to be exactly the true segment number, because we wanted to allow the model to make occasional errors at some frames (i.e., break a segment) without being forced to connect another two segments at other frames. In this setting, we observed that the inferred Viterbi segmentations obey the specified constraints on the number of segments. Furthermore, with this constraint, THEMIS's overall accuracy improved in most of the simulations (Table 15).

References

[1] Gavin Ha, Andrew Roth, Jaswinder Khattra, Julie Ho, Damian Yap, Leah M Prentice, Nataliya Melnyk, Andrew McPherson, Ali Bashashati, Emma Laks, et al. TITAN: inference of copy number architectures in clonal cell populations from tumor whole-genome sequence data. *Genome Research*, 24(11):1881–1893, 2014.

[2] Kristian Cibulskis, Michael S Lawrence, Scott L Carter, Andrey Sivachenko, David Jaffe, Carrie Sougnez, Stacey Gabriel, Matthew Meyerson, Eric S Lander, and Gad Getz. Sensitive detection of somatic point mutations in impure and heterogeneous cancer samples. *Nature Biotechnology*, 31(3):213–219, 2013.

[3] Stefano Colella, Christopher Yau, Jennifer M Taylor, Ghazala Mirza, Helen Butler, Penny Clouston, Anne S Bassett, Anneke Seller, Christopher C Holmes, and Jiannis Ragoussis. Quantisnp: an objective bayes hidden-markov model to detect and accurately map copy number variation using snp genotyping data. *Nucleic Acids Research*, 35(6):2013–2025, 2007.
